# Diagnosis: Congenital Dyserythropoietic Anemia Type 2 Due to Compound Heterozygote Mutation in SEC23B Gene

**DOI:** 10.4274/tjh.2014.0478

**Published:** 2015-08-01

**Authors:** Fatih Demircioğlu, Mustafa Erkoçoğlu, Mustafa Dilek, Mervan Bekdaş, Sevil Göksügür, Semra Büyükkorkmaz, Seher Açar

**Affiliations:** 1 Abant İzzet Baysal University Faculty of Medicine, Department of Pediatrics, Division of Pediatric Hematology, Bolu, Turkey; 2 Abant İzzet Baysal University Faculty of Medicine, Department of Pediatrics, Bolu, Turkey

**Keywords:** Anemia, Congenital dyserythropoietic anemia type 2, SEC23B gene

## QUIZ IN HEMATOLOGY

**Figure 1:**
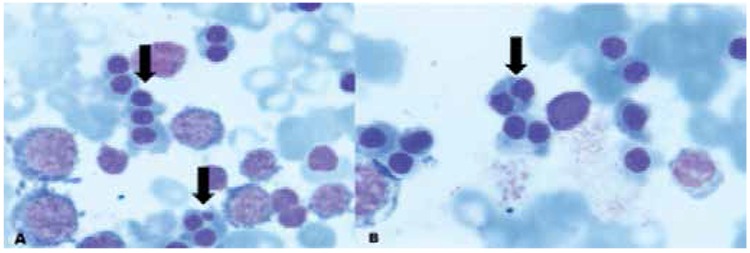
A and B: Marked dyserythropoiesis, nuclear bridging, binucleation, and multinucleation of the erythroid precursors.

A 14-year-old female patient was admitted to our hospital because of paleness and icterus. She had been investigated at different centers for chronic anemia, icterus, and splenomegaly since age 6. She had no history of blood transfusion, drug usage, or fever. There was no consanguinity between parents, and our patient’s sister had similar clinical findings. On physical examination she was pale and icteric and she had markedly enlarged liver and spleen, 3 cm and 8 cm below the costal margins, respectively. Respiratory, cardiovascular, musculoskeletal, and other examinations were normal. Informed consent was obtained. The hemogram revealed Hb: 8.0 g/dL, RBC: 2.59x1012/L, Hct: 24.5%, MCV: 94.6 fL, RDW: 17.1, WBC: 7.65x109/L, and platelets: 383x109/L. Reticulocyte count was 3.4% and peripheral blood smear revealed anisocytosis and poikilocytosis. The liver and kidney function test results were within normal limits. Serum total and indirect bilirubin and LDH levels were 5.22 mg/dL, 0.46 mg/dL, and 545 IU/L respectively. Direct Coombs test was negative. Hemoglobin electrophoresis, pyruvate kinase and G6PD, vitamin B12, and folic acid levels were normal. She was immune to HBV infection and her serological tests were negative for HCV and HIV. Bone marrow examination under light microscopy showed erythroid hyperplasia, with binucleated or multinucleated and megaloblastic normoblasts (Figures 1A and 1B):

A clinical diagnosis of congenital dyserythropoietic anemia (CDA) type 2 was established after exclusion of other causes associated with dyserythropoiesis and ineffective erythropoiesis, including thalassemia syndromes, vitamin B12 and folate deficiencies, myelodysplastic syndrome, and sideroblastic anemia. Molecular analyses for CDA type 2 revealed compound heterozygote mutations in the SEC23B gene (c.325G >A, p.Glu109Lys in exon 4 and c.938 G >A, p.Arg313His in exon 8). 

CDA is a heterogeneous group of rare hereditary disorders of ineffective erythropoiesis and dyserythropoiesis characterized by morphologically abnormal erythroid precursors in the bone marrow. Non-erythroid hematopoietic cell morphology is normal [1]. Type 2 (CDA 2), the most common type, is autosomal recessive and typically shows marked dyserythropoiesis characterized by binucleated and multinucleated normoblasts with erythroid hyperplasia [2,3,4]. Most patients with CDA 2 show variable degrees of anemia, usually have jaundice, and may have hepatosplenomegaly. Our patient was diagnosed as having CDA type 2 by the presence of anemia, icterus, hepatosplenomegaly, binucleated and trinucleated erythroid precursors in the bone marrow, and positive SEC23B mutation.

**Conflict of Interest Statement**

The authors of this paper have no conflicts of interest, including specific financial interests, relationships, and/or affiliations relevant to the subject matter or materials included.
